# Expression of Female Sex Hormone Receptors, Connective Tissue Growth Factor and HER2 in Gallbladder Cancer

**DOI:** 10.1038/s41598-020-58777-y

**Published:** 2020-02-05

**Authors:** Beata Hryciuk, Rafał Pęksa, Michał Bieńkowski, Bartosz Szymanowski, Barbara Radecka, Kamil Winnik, Jolanta Żok, Natalia Cichowska, Mariola Iliszko, Renata Duchnowska

**Affiliations:** 1 Department of Oncology, Military Institute of Medicine, Warsaw, Poland; 2 Mazovian Center for Lung Diseases and Tuberculosis, Division III in Otwock, Otwock, Poland; 3 Pathology Department, Medical University of Gdańsk, Gdańsk, Poland; 4 University of Opole, Institute of Medical Science, Opole, Poland; 5 Pathology Department Provincial Specialist Hospital, Słupsk, Poland; 6 Department of Chemotherapy, Center of Pulmonology and Chemotherapy, Szklarska Poręba, Poland; 7 Department of Oncology and Radiotherapy, Medical University of Gdańsk, Gdańsk, Poland; 8 Department of Biology and Medical Genetics, Medical University of Gdańsk, Gdańsk, Poland

**Keywords:** Gastrointestinal cancer, Tumour biomarkers, Biomarkers, Gastroenterology, Medical research, Oncology, Pathogenesis, Risk factors

## Abstract

Gallbladder cancer (GBC) is a highly malignant tumor with poorly understood etiology. An insight into phenotypic features of this malignancy may add to the knowledge of its carcinogenesis and pave the way to new therapeutic approaches. We assessed the expression of female sex hormone receptors (ERα, ERβ, PR), connective tissue growth factor (CTGF) and HER2 in GBC, and adjacent normal tissue (NT), and determined their prognostic impact. Immunohistochemical (IHC) expression of all biomarkers was performed in formalin-fixed, paraffin-embedded specimens in 60 Caucasian GBC patients (51 women and 9 men). ERβ, cytoPR and CTGF expression were found in 89%, 27%, 91% of GBC, and in 63%, 87%, 100% of NT, respectively. No ERα expression was found in GBC and NT. Strong (3+) HER2 expression by IHC or *HER2* amplification was seen in five GBC (10.4%). A positive correlation was found between HER2 and CTGF and ERβ expression in GBC and matched NT. In the multivariate analysis, patient age >70 years, tumor size and ERβ expression in GBC was highly predictive for OS (p = 0.003). The correlation between HER2, CTGF and ERβ expression in GBC and NT may indicate the interaction of these pathways in physiological processes and gallbladder pathology.

## Introduction

Gallbladder cancer (GBC) is an aggressive malignant tumor originating from epithelial cells of the mucous membrane. The etiology of GBC is poorly understood, and its global prevalence is characterized by considerable regional variations^1–4^. The highest incidence is reported in Chile, India, Pakistan, Bolivia, Central Europe, Israel, and in Native Americans and Americans of Mexican origin^1–4^. The risk of developing GBC is higher in patients with chronic inflammatory processes caused by gallstones with resulting calcification of the gallbladder wall (“porcelain gallbladder”), and with infections such as *Salmonella typhi* or *Salmonella paratyphi*^3–5^. GBC is about two to six times more common in women compared to men, and its incidence steadily increases with age^1–5^.

Estrogens are key signaling molecules that regulate various physiological processes and play a major role in many pathological conditions, such as hormone-dependent cancers. Expression of female sex hormone receptors in GBC has been analyzed in a few studies including mainly Asian populations, and provided inconsistent results^6–14^. Several small studies demonstrated overexpression of human epidermal growth factor receptor type 2 (HER2) and *HER* gene amplifications or mutations^15–27^. Connective Tissue Growth Factor (CTGF) was found to be expressed in various stages of the GBC carcinogenesis^28^. GBC carries a poor prognosis and most patients succumb to their disease. An insight into phenotypic features of this tumor may add to the knowledge of its carcinogenesis and pave the way to new therapeutic approaches. In the present study we investigated the expression of female sex hormone receptors: estrogen receptor alpha (ERα), ER beta (ERβ) and progesterone (PR), as well as CTGF and HER2 in a relatively large group of Caucasian GBC patients. Additionally, we assessed prognostic value of these biomarkers.

## Results

### Patient characteristics

The study group included 60 GBC Caucasian patients (51 women and 9 men). The average age of patients was 67 years (range, 31–97 years), median BMI was 26.2 (range, 17.7–4.3) and in 35 patients (58%) GBC coexisted with gallstones. The simple cholecystectomy or *en bloc* resection of gallbladder with segments IVb and V of the liver, with or without regional lymph node dissection was performed in 49 patients (82%). After a median follow-up of 8 months (range 0–167), 31 patients (52%) developed relapse, 29 of whom (48%) received palliative chemotherapy. All cases were diagnosed as adenocarcinoma, not otherwise specified. Pathologic stages T1, T2, T3 and T4 were found in 13 (22%), 23 (38%), 14 (23%) and 4 (6.7%) of patients, respectively, and in 6 patients (10%) pT status was not determined. Pathologic N0 and pN1 stages were seen in 11 (18%) and 13 (22%) of patients, respectively, and in 36 patients (60%) pN status was not determined. High (G3), intermediate (G2), and low-grade (G1) adenocarcinoma was diagnosed in 11 (18%), 29 (48%), and 11 (18%) of cases, respectively, and in nine patients (15%) grade was not determined (Table 1).

**Table 1:** Patient characteristics.

Variable	n (%)
Age at GBC diagnosis (years)	60 (100)
Mean	67
Range	61–76.5
Sex	60 (100)
Women	51 (85)
Men	9 (15)
Body mass index (kg/m^2^)	34 (57%)
Mean	26.2
Range	17.7–45.3
Gallstones	60 (100)
No	17 (28.3)
Yes	35 (58.3)
Unknown	8 (13.4)
Histology	60 (100)
Adenocarcinoma, NOS	60 (100)
Grade (G)	60 (100)
G1	11 (18.3)
G2	29 (48.3)
G3	11 (18.3)
G4	0
Unknown	9 (15.1)
pT stage	60 (100)
1	13 (21.7)
2	23 (38.3)
3	14 (23.3)
4	4 (6.7)
Unknown	6 (10.0)
pN stage	60 (100)
0	11 (18.3)
1	13 (21.7)
Unknown	36 (60.0)
Surgery	60 (100)
Resection^a^	49 (82.0)
Diagnostic laparoscopy	6 (10.0)
Unknown	5 (8.0)
Disease recurrence	60 (100)
No	29 (48.3)
Yes	31 (51.7)
Palliative chemotherapy	60 (100)
No	14 (23.3)
Yes	29 (48.3)
Unknown	17 (28.4)

^a^Including simple cholecystectomy or *en bloc* resection of gallbladder with segments IVb and V of the liver with or without regional lymph nodes.

### Biomarker expression

**Figure 1:**
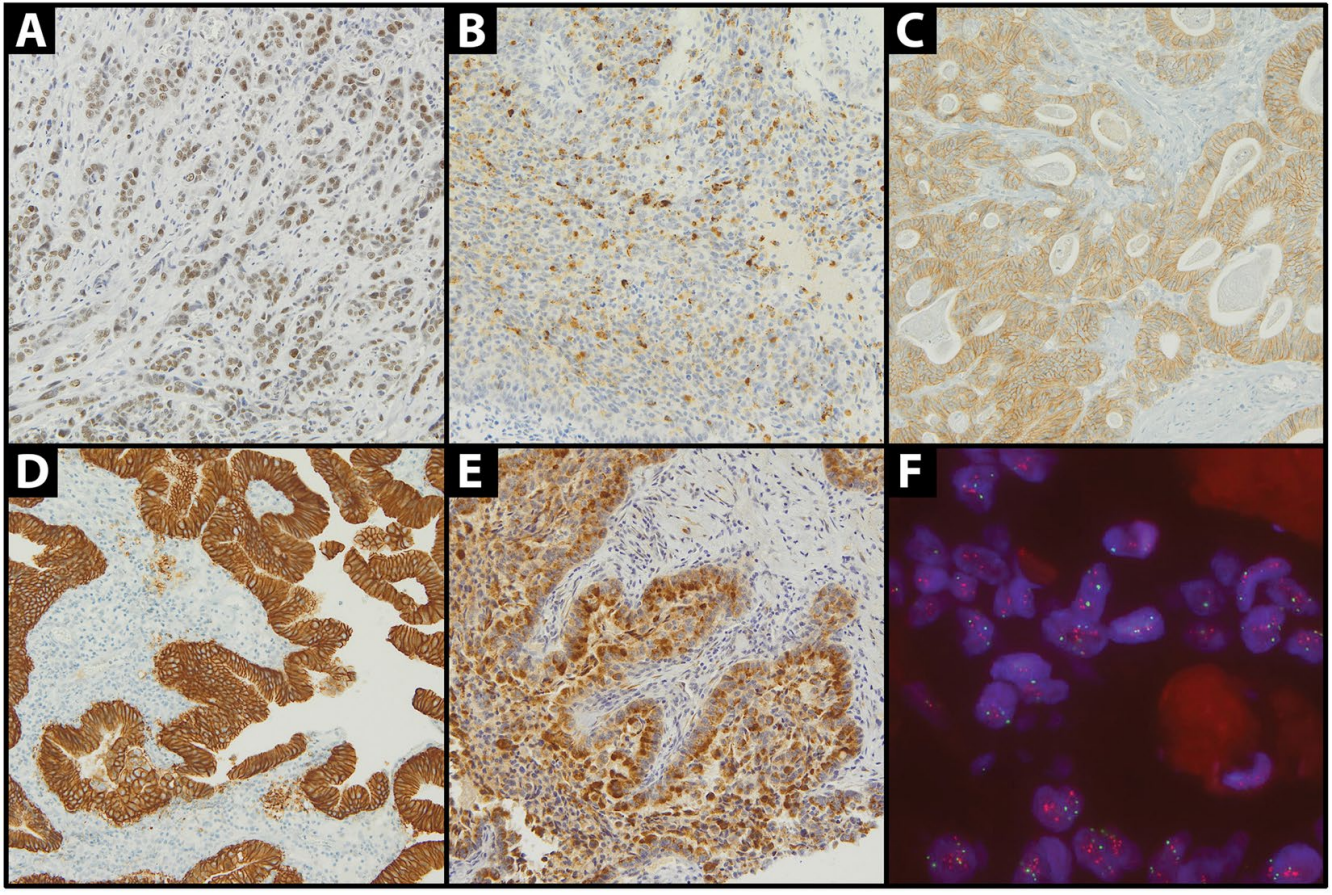
Immunohistochemical expression (magnification X20): (**A**) ERβ; (**B**) cytoPR; (**C**) HER2 (2+); (**D**) HER2 (3+); (**E**) CTGF. and (**F**) FISH *HER2* gene amplification.

ERβ, cytoPR and CTGF expression was found in 40 (89%), 15 (27%) and 48 (91%) cases of GBC and in 22 (63%), 34 (87%) and 38 (100%) of NT, respectively (Table 2). No ERα or nuclear PR expression was found in GBC and in adjacent NT. Loss of tissue spots for ERβ, cytoPR, HER2 and CTGF analysis was 15 (25%), 5 (8.3%), 12 (20%), 7 (11.7%) in GBC and 25 (41.7%), 21 (35%), 21 (35%), 22 (36.7%) in adjacent NT, respectively. Compared to NT, GBC specimens showed higher expression of ERβ (p < 0.01), and lower expression of HER2 (H-score) and cytoPR (p < 0.01 and p < 0.01, respectively). IHC HER2 2+ expression was found in 6 (13%) and 6 (15%) of GBC and NT, respectively. IHC HER2 3+ or 2+ expression and *HER2* amplification was seen in five GBC (10.4%; Fig. 1C,D,F). Intermediate HER2 expression (HER2 2+) was also found in six normal NT, none of which with *HER2* gene amplification by FISH (Table 2). There was no significant difference in the expression of CTGF between GBC and NT (p = 0.16). A positive correlation was found between HER2 and CTGF expression in GBC and matched NT (p = 0.003 and p < 0.001, respectively). ERβ and HER2 expression in GBC and in matched NT showed a borderline correlation (p = 0.056 p = 0.052, respectively). Expression of cytoPR in NT was higher in patients with gallstones (p = 0.04). There was no association between ERβ, cytoPR, HER2 and CTGF expression in GBC and the presence of gallstones (p = 0.39; p = 1.0; p = 1.0; p = 0.17; p = 1.0, respectively).

**Table 2:** Biomarkers expression in the GBC and in adjacent normal tissue (GBC: gallbladder cancer, NT: adjacent normal tissue).

Biomarker	GBC (N, %)		NT (N, %)	
	Positive	Negative	Positive	Negative
ERβ	40 (89%)	5 (11%)	22 (63%)	13 (37%)
cytoPR	15 (27%)	40 (73%)	34 (87%)	5 (13%)
HER2 (2+) IHC	6 (13%)	42 (87%)	6 (15%)	33 (85%)
HER2 (3+) IHC or FISH positive	5 (10.4%)	43 (89.6%)	0 (0%)	39 (100%)
CTGF	48 (91%)	5 (9%)	38 (100%)	0 (0%)

### Clinical outcomes

**Figure 2:**
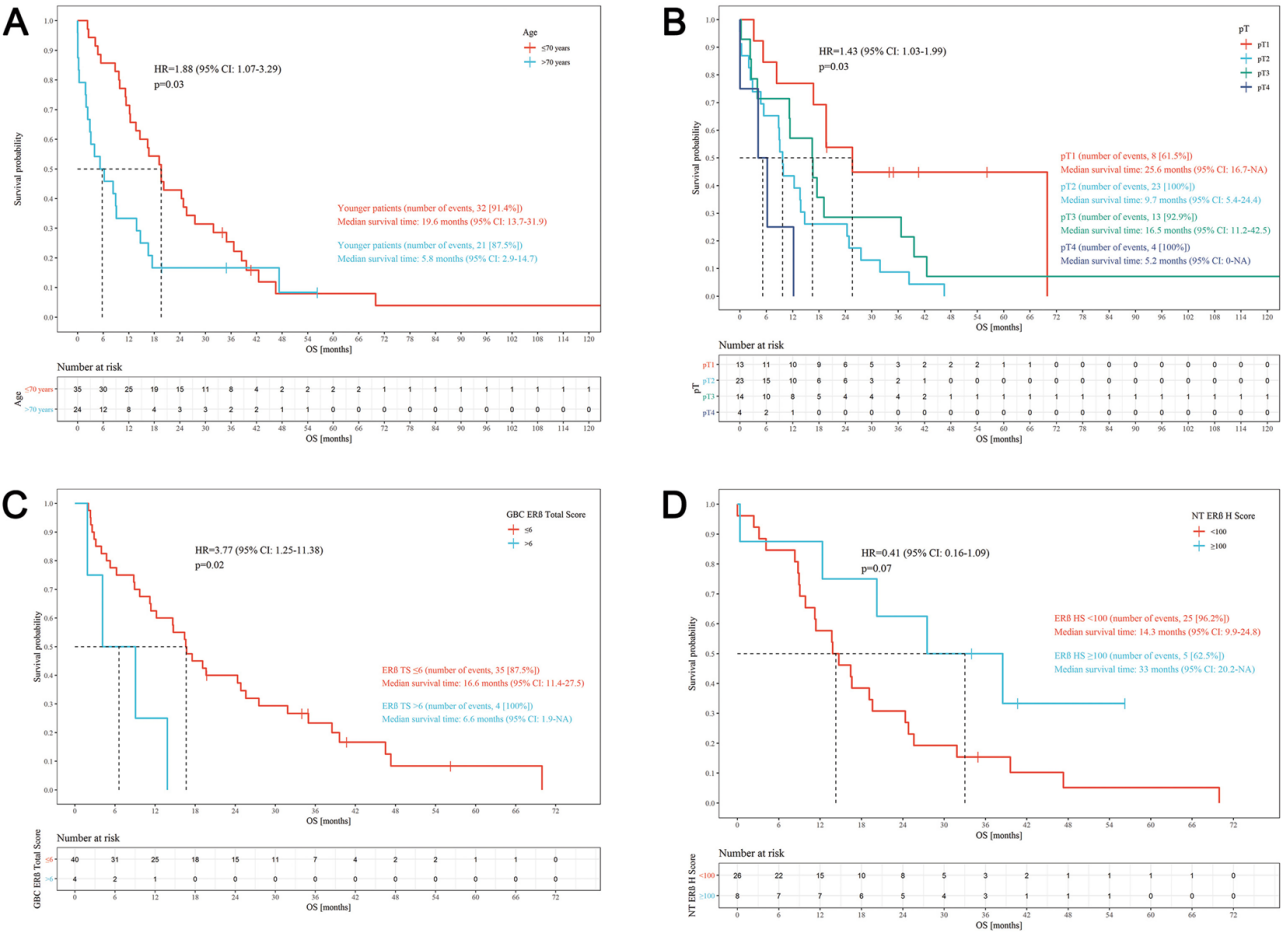
Kaplan-Meier overall survival curves. (**A**) Age ≤70 vs >70 years; (**B**) pT stage; (**C**) ERβ expression in GBC ≤6 vs >6; (**D**) ERβ expression in normal adjacent tissue H-score <100 vs ≥100.

Median overall survival was 13.6 months (range, 1–167). In the univariate analysis, age above 70 years (HR = 1.88; 95%CI 1.07–3.29; p = 0.03) and pT stage (HR = 1.43; 95%CI 1.03–1.99; p = 0.03) were correlated with shorter OS (Fig. 2A,B). Expression of ERβ in GBC was correlated with shorter (p = 0.02), and in NT with longer OS (p = 0.03) (Fig. 2C,D). HER2 expression in GBC did not impact OS (p = 0.12). In the multivariate analysis, shorter OS was correlated with age above 70 years, higher pT stage, and expression of ERβ in GBC (p = 0.003).

## Discussion

We have performed a comprehensive analysis of female sex hormone receptors, HER2 and CTGF in GBC and in adjacent NT in a relatively large group of Caucasian patients. The lack of ERα expression in GBC and in adjacent NT confirms the results of studies published after 2007^12–14^. ER expression demonstrated in some earlier reports was likely due to nonspecific staining with antibodies involving both ERα and ERβ receptors^6–11^. In these studies ER expression was demonstrated in both metaplasia, and GBC, irrespective of its differentiation^6,7,9^. The prognostic value of ER expression in GBC is contradictory. Two studies from India suggested that the expression of ER and PR in GBC does not have an impact on the prognosis^12,13^. In our study ERβ expression in GBC was correlated with a shorter OS, and in NT with a longer OS. This finding is intriguing but may be incidental owing to multiple comparisons. In some malignancies, including breast, ovarian and prostate cancers, ERβ plays a suppressive and anti-proliferative roles^29,30^. In contrast to ERβ, expression of cytoPR was higher in NT compared to GBC, and was associated with the co-existence of gallstones. Additionally, cytoPR expression was negatively correlated with CTGF expression. A few studies demonstrated PR expression (including cytoPR) in GBC^6,11,12^. In the Baskaran *et al*. study^11^ PR was more often expressed in neoplastic compared to benign lesions, and in the Nakamura *et al*. study^6^, PR expression was lower in the metaplasia and high-grade GBC. Some studies did not demonstrate PR expression in GBC, which may be related to the use of antibodies detecting only nuclear PR expression (PR-A isoform), whereas isoform B (PR-B) is also expresses in the cell cytoplasm^13,14,31^. The different functions of three PR isoforms (A, B, and C) are well recognized in breast cancer^32–34^. PR-B activates expression of progesterone-dependent genes by palindromic-progesterone-response DNA elements related to the metabolism of sex hormones^32–34^.

High expression of ERβ GBC found in this study supports a possible role of anti-estrogen therapy in GBC, probably with a different approach than in breast cancer though. In obese postmenopausal women, adipose tissue is the main source of estrogen biosynthesis, and this hormone has been shown to increase cholesterol level in bile and decrease gallbladder contractility^35^. Likewise, physiological processes in premenopausal women, such as menstrual cycle phase and pregnancy, or contraceptive use, are accompanied by changes of gallbladder functions and increased gallstone formation^36,37^. A recent study postulated that cholesterol gallstones in women are related to differences in liver cholesterol metabolism in response to estrogen, a process mediated by up-regulating of the *ESR1* (ERα coding gene) expression^38^. In other study *ESR1* polymorphic variants: IVS1-397C > T, ESR1 IVS1-351A > G and ESR2-789 A > C correlated with GBC risk, mediated through gallstone dependent pathway^39^.

Tamoxifen is the oldest and most-prescribed selective estrogen receptor modulator in breast cancer patients. A Turkish study demonstrated increased risk of gallstone formation in postmenopausal breast cancer patients administered tamoxifen^40^, but this effect was not confirmed in another study performed in India^41^. Interestingly, patients with *ESR1-*mutated breast cancer showed better response to fulvestrant, a hormonal protein degrader, compared to aromatase inhibitors^42^. Further research is warranted to assess the fulvestrant activity in GBC.

The present study did not show different CTGF expression in GBC and adjacent NT, or the prognostic value of this biomarker. The role of CTGF in GBC progression and its favorable prognostic impact was earlier reported in a Chilean study^28^. This effect may be attributed to the stromal response to the neoplastic process in an autocrine or paracrine manner^28^. Inconsistent results of both studies may be due to different etiology of GBC in the Latin American and Caucasian populations^3,4,6^.

HER2 overexpression or gene amplification occur in 12–15% of GBC^15,20,22–25^. In our study, using the breast cancer criteria, IHC 2+ or 3+ expression was found in 15% of cases, more than a half of which (8.3%) showed a true HER2 positivity (IHC 3+ expression or FISH amplification). Aberrant HER family signaling may be important in the development and progression of GBC^15,20^. Some studies reported adverse prognostic impact of HER2 expression^16,23–26,43^, but others, including ours, did not show such correlation^27,44^. A few studies have investigated anti-HER2 therapy in advanced GBC^45–49^. This approach was also attempted in bilary tract cancer (BTC) patients. Two phase II studies in unselected BTC patients did not show lapatinib activity^45,46^. In the MyPathway trial including seven HER2-positive BTC patients treated with the combination of the anti-HER2 antibodies, trastuzumab and pertuzumab, the objective response rate was 29%^47^. In the NCT02675829 clinical trial, the response rate for ado-trastuzumab emtansine in *HER2* amplified BTC patients was 17%^48^. Recently, a basket trial showed the activity of a pan-HER tyrosine kinase inhibitor neratinib in *HER2*-mutant BTC patients^49^. In our study expression of HER2 and CTGF in GBC was positively correlated with their expression in surrounding NT. This finding may suggest a connection between these pathways in both physiological and pathological processes of the gallbladder. For example, in breast cancer, there is a progestin-independent relationship between the pathways for steroid receptors and growth factor receptors^50^.

Similarly to other GBC studies, age over 70 years and higher pT stage adversely impacted OS^1–5^.

Our study contributes to the current knowledge on the biology of GBC, but owing to its retrospective nature and the relatively small group of patients, should be interpreted cautiously. In recent years, somatic profiling with next-generation sequencing has identified several genes, including *TP53, SMAD4* and *KRAS*, which seem to play a role in GBC carcinogenesis^51–53^. At present, a number of agents targeting new pathways are being investigated in clinical trials in GBC patients. Our tissue material and clinical database may be exploited in future scientific projects.

## Materials and Methods

### Study population

This study was approved by the Institutional Review Board of the coordinating center, the Military Institute of Medicine in Warsaw, Poland. The patients were diagnosed and underwent surgery between 2004 and 2016 in four oncology centers in Poland. Demographic, clinicopathologic, and clinical follow-up data were extracted from medical records. All data were coded to secure full protection of personal information, therefore, patient consent was not sought. All research was performed in accordance with relevant guidelines and regulations.

### Immunohistochemical analysis

The starting material from each patient was an archival formalin-fixed, paraffin-embedded (FFPE) surgical specimen of the primary GBC. The pathologic diagnosis was confirmed by a board-certified pathologist (RP) who reviewed FFPE tissue sections stained with hematoxylin and eosin. A representative paraffin block from each specimen was chosen for immunohistochemical analysis (IHC). The two biopsy specimens of GBC and surrounding NT (“tissue core”) were placed on the previously prepared tissue-free paraffin blocks (“recipients”). Tissue microarrays were constructed using Manual Tissue Arrayer I by Beecher Instruments (MTAI, K7 BioSystems). IHC was performed on 4 μm thick tissue microarray sections. The staining was conducted according to the manufacturers’ protocols (Table 3). ERα, ERβ and PR were evaluated in the cell nuclei or/and cytoplasm. The occurrence of nuclear and/or cytoplasmatic ERα, ERβ, PR reaction in at least 1% cells was considered a positive reaction. CTGF expression was evaluated in the cytoplasm and cell membrane, and HER2 expression in the cell membrane. For all biomarkers the intensity of staining was defined as weak (1), moderate (2), or strong (3). The H-score was calculated for each biomarker by the formula: 3 × % strong cellular staining (cytoplasmic, nuclear and/or membranous) + 2 × % moderate staining + 1 × % weak staining. This made a range of 0–300. Additionally, ERα, ERβ and PR were scored by the Allred method. This system is graded on a scale of 0 to 8, with 0 indicating a completely negative result and 2 to 8 used as a means of semiquantifying the immunoreactivity^54^. Based on breast cancer criteria for HER2-positivity only samples showing strong expression (scored 3 IHC), defined as uniform and intense membrane staining of at least 10% of invasive tumor cells, were considered positive. The samples showing intermediate expression (scored 2 IHC) were subjected to additional analysis of HER2 gene copy number using fluorescence *in situ* hybridization (FISH). Gene amplification by FISH was defined as a FISH ratio (HER2/centromeric probe for chromosome 17 ratio) of greater than 2.0. FISH-positive patients were considered HER2-positive^55^. Figure 1 shows examples of positive control staining for ERβ, cytoPR, HER2 (2+), HER2 (3+), CTGF and *HER2* gene amplification.

**Table 3:** Antibodies, dilutions and methods of evaluation.

Target	Manufacturer	Dilution	Epitope retrival	Incubation	Control tissue	Method of evaluation
ERα	DAKO; anti-human; rabbit clone EP1	RU	HIER	20′	BC	SQ
ERβ	Abcam; anti-human; rabbit clone EPR3778; ab133467	1:70	HIER	night incubation	BC	SQ
PgR	DAKO; anti-human; mouse clone 636	RU	HIER	20′ +linker mouse 15′	BC	SQ
HER2	Ventana; rabbit clone 4B5	RU	Epitope retrival in machine	20′	BC	SQ
CTGF	Santa Cruz, California; goat sc-14939	1:100	HIER	60′	SM	SQ

RU: ready to use; HIER: Heat-Induced Epitope Retrieval; SQ: semiquantitative; BR: breast cancer; SM: smooth muscle.

### Statistical analysis

The statistical analysis was conducted using statistical environment R, rev. 3.4.3., on the basis of data contained in a study-dedicated database. This analysis included all available clinical and pathological variables. Expression of individual biomarkers was compared using the intraclass correlation coefficient (ICC), assuming kappa <0.4 as weak, ≥0.4 as sufficient, ≥0.6 as good and ≥0.75 as optimal correlation, and with Kendall tau test — ICC package. The Mann-Whitney-Wilcoxon test was used to compare biomarker expression between GBC and surrounding NT. Overall survival (OS) was computed using the Kaplan-Meier method, starting from GBC diagnosis to the date of death or the last follow up. Univariate and multivariate analyses were performed using the log-rank test, Wilcoxon test, and Cox proportional hazard and logistic regression.
